# Impact of romantic love on obsessive-compulsive disorder phenotypes

**DOI:** 10.1192/j.eurpsy.2025.1716

**Published:** 2025-08-26

**Authors:** D. Marazziti, R. Gurrieri, N. Schulz Bizzozzero Crivelli, M. Gambini, A. Arone, S. Palermo, L. F. D. C. Fontenelle

**Affiliations:** 1 Department of Clinical and Experimental Medicine; 2 Department of Surgical-Medical and Molecular Pathology and Critical Care Medicine, University of Pisa, Pisa, Italy; 3 Institute of Psychiatry of Federal University of Rio de Janeiro, Rio de Janeiro, Brazil

## Abstract

**Introduction:**

In recent years, multiple observational studies have been conducted to investigate the hypothesis of a correlation between romantic love (RL) and the phenotypic expression of obsessive-compulsive disorder (OCD).

**Objectives:**

Our study aimed to evaluate the impact of RL on the clinical expression of OCD. Attention was specifically focused on investigating the onset of two OCD phenotypes with distinct characteristics, based on whether the subjects were at the onset of a romantic relationship or had experienced a romantic break-up, also considering the possible correlations with different clinical aspects and socio-demographic variables.

**Methods:**

Our sample includes a total of 212 subjects with OCD recruited among outpatients at the University Psychiatric Clinic of Pisa, Italy, and the Federal University of Rio de Janeiro, Brazil. The following instruments were employed for psychometric assessments: the Structured Clinical Interview for DSM-5 (SCID-5), the Yale OCD Natural History Questionnaire, and the Yale-Brown Obsessive-Compulsive Scale (Y-BOCS). The study participants were then divided into two groups (love-precipitated [LP-OCD] and break-up OCD [BU-OCD]) according to the romantic factor that was deemed responsible for the onset of OCD. An appropriate statistical analysis was applied.

**Results:**

The average age of onset of OCD was significantly different between the two groups, which may reflect a vulnerability of the brain’s maturational stages in young individuals who are at risk for OCD. A trend towards three types of obsessions and compulsions (aggression, sexual/religious and symmetry, ordering, and rearrangement) in the BU-OCD group emerged, which may reflect some normal features of a romantic relationship. However, total Y-BOCS obsessions and compulsions subscale scores were similar, indicating an overall severe clinical picture.

**Conclusions:**

Despite some limitations, our results suggest that different stages of RL might influence some characteristics of OCD, namely age at onset and some specific dimensions, but would not appear to interfere with the overall severity of the disorder. These results should encourage further research on the topic to learn more about the characteristics of these individuals and to better understand how the most natural experience of humankind, that is love, may represent a vulnerability factor towards the onset and some features of OCD, similarly to other mental disorders, where the evidence is currently strongest.

**Disclosure of Interest:**

None Declared

